# Transcriptomic and Metabolomic Analyses Reveal the Attenuating Role of Cordycepin and *Cordyceps militaris* Extract on Acute Liver Injury Induced by LPS in Piglets

**DOI:** 10.3390/ani14192873

**Published:** 2024-10-05

**Authors:** Ding Tan, Endian Li, Shijie Xiong, Yue Sun, Wenbo Cheng, Yong Su, Yang Lu

**Affiliations:** 1 Laboratory of Gastrointestinal Microbiology, Jiangsu Key Laboratory of Gastrointestinal Nutrition and Animal Health, College of Animal Science and Technology, Nanjing Agricultural University, Nanjing 210095, China; 2022105067@stu.njau.edu.cn (D.T.); 15122310@stu.njau.edu.cn (E.L.); 2022105066@stu.njau.edu.cn (S.X.); 12122127@stu.njau.edu.cn (W.C.); 2 Institute of Animal Husbandry and Veterinary Science, Shanghai Academy of Agricultural Sciences, Shanghai 201106, China

**Keywords:** piglets, *Cordyceps militaris* extract, cordycepin, liver injury, metabolomics and transcriptomics

## Abstract

**Simple Summary:**

Lipopolysaccharide (LPS), an endotoxin derived from the outer membrane of Gram-negative bacteria, is one of the causes of acute liver injury. *Cordyceps militaris* extract (CME) contains many bioactive compounds, mainly cordycepin (CPN). This study aimed to investigate the possible mechanisms underlying the amelioration of LPS-induced acute liver injury in piglets by CME or CPN supplementation using transcriptomic and metabolomic analyses. We found that CPN or CME supplementation significantly decreased the C-reactive protein level and improved liver tissue pathology after LPS injection. Transcriptomic and metabolomic results showed that CME or CPN supplementation could attenuate liver injury by downregulating LPS-induced liver inflammation-related pathways and by modulating the levels of immune response-related metabolites. The present study reveals the immunomodulatory effects of CPN and CME against LPS-induced liver injury.

**Abstract:**

*Cordyceps militaris* extract (CME) contains many bioactive compounds, mainly cordycepin (CPN). This study aimed to investigate the possible mechanisms underlying the amelioration of LPS-induced acute liver injury in piglets by CME or CPN supplementation using multi-omics analysis. Twenty-four weaned piglets were randomly distributed into 4 groups (*n* = 6): the control and LPS groups were fed basal diets; the CPN + LPS (CPN-LPS) and CME + LPS (CME-LPS) groups were fed the basal diets supplemented with CME or CPN. The results showed that CPN or CME supplementation significantly decreased the C-reactive protein level (*p* < 0.05) and improved liver tissue pathology to prevent acute liver injury after LPS treatment. Compared with LPS, the transcriptomic analysis indicated that CPN supplementation significantly downregulated cell adhesion molecules, while CME supplementation significantly downregulated inflammatory mediator regulation of TRP channels, complement and coagulation cascades and cytokine-cytokine receptor interaction. The metabolomic results showed that CPN or CME supplementation significantly reduced disease biomarker of bicyclo-prostaglandin E2, and increased levels of deoxyinosine and 3-hydroxyanthranilic acid (*p* < 0.05). The combined transcriptome and metabolome helped identify two metabolites PC 34:2 and PC 36:0, which may be associated with the restoration of liver cell morphology. In conclusion, CPN and CME could attenuate LPS-induced acute liver injury by regulating immune-related genes and metabolites. This study elucidates the potential protective mechanism of CPN or CME supplementation against acute liver injury.

## 1. Introduction

Lipopolysaccharide (LPS), an endotoxin derived from the outer membrane of Gram-negative bacteria, is one of the causes of acute liver injury [1]. Studies have proved that LPS, as an inducer, can stimulate the body’s immune system, trigger oxidative stress and inflammatory responses in the body or cells, and even cause liver cell death, thus leading to liver dysfunction [2]. Specifically, a study conducted by Duan, et al. [3] indicated that LPS could cause liver damage in piglets, as evidenced by the upregulation of pro-inflammatory factors such as TNF-α (tumour necrosis factor-α) and IL-6 (interleukin-6), elevated levels of MPO (myeloperoxidase) and MDA (malondialdehyde), and impaired mitochondrial morphology. Additionally, Zhao, et al. [4] demonstrated that LPS caused pathological damage to liver tissue in piglets, increased serum levels of alanine aminotransferase (ALT) and aspartate aminotransferase (AST), and induced liver cell apoptosis. The liver plays a pivotal role in metabolic and detoxification processes, acting as the final barrier that protects the body from exogenous toxic substances [5,6]. Acute liver injury caused by bacteria may further develop into liver failure, particularly in young livestock, with a high mortality rate [7]. LPS has been extensively employed in the construction of models for acute liver injury, the investigation of the pathological mechanisms of acute liver injury and the development of pharmacological or nutritional interventions [8]. Therefore, acute liver injury by LPS can be modelled to investigate how to prevent the occurrence of acute liver injury in animal husbandry.

*Cordyceps militaris*, an important edible and medicinal fungus with a variety of biological functions, including immune regulation, hypolipidemic, anti-tumour, anti-aging, etc., is widely known to contain a number of active ingredients, including cordycepin, polysaccharides, peptides, and alkaloids [9,10,11]. Cordycepin (CPN), as one of its main active ingredients, has been extensively reported in the treatment of a range of liver diseases [12,13,14,15]. For example, a study showed that cordycepin alleviated hepatic steatosis, inflammation, liver injury and fibrosis in mice subjected to metabolic stress by activating the AMPK signalling pathway, which in turn mitigated non-alcoholic steatohepatitis [13]. Moreover, Li, et al. [14] demonstrated that cordycepin protected against D-galactosamine (GalN)/LPS-induced acute liver injury in mice by improving liver function indices, inhibiting hepatocyte apoptosis and necrosis, and reducing hepatic neutrophil and macrophage infiltration. A similar study showed that a *Cordyceps mycelia* extract (CME), the main active ingredient of which is cordycepin, plays a beneficial role in the prevention and treatment of dimethylnitrosamine (DMN)-induced portal hypertension in cirrhotic rats [16]. Our previous study has shown that the addition of cordycepin or *Cordyceps militaris* extract can effectively regulate the intestinal microbial community of piglets and inhibit the inflammatory response and oxidative stress induced by LPS [11]. However, studies on cordycepin or *Cordyceps militaris* extract to alleviate LPS-induced acute liver injury are still scarce and the underlying mechanisms are not fully understood and require further investigation.

The development and application of multi-omics analysis have provided novel methodologies for the comprehension of intricate molecular mechanisms [17,18]. Transcriptomics research can assist in the comprehension of gene expression processes under specific conditions, thereby facilitating the investigation of the functions of genes exhibiting alterations in expression [19]. Metabolomics can dynamically monitor compounds produced by internal and external factors in the body and can be used to assess the effects of environmental pollutants and drugs, and infer their potential mechanisms of action [20,21]. Therefore, in this study, piglets were fed the basal diet supplemented with CPN or CME and the acute liver injury model was established by intraperitoneal injection of LPS to verify the hypothesis that cordycepin could alleviate acute liver injury induced by LPS. Then, further analysis through transcriptomics and metabolomics was intended to elucidate the potential mechanisms.

## 2. Materials and Methods

### 2.1. Preparation of Cordyceps Militaris Extract and Cordycepin

Referring to our previous research [11], the residues of *Cordyceps militaris* were purchased from Jiangsu Kangneng Bioengineering Co., Ltd. in Yangzhou, China, and then the active substances (CME) containing 1% cordycepin were extracted through a series of steps such as grinding, water extraction, solid–liquid separation, vacuum concentration and spray drying. After that, the active substance was further purified and dried to obtain CPN with a content of 90%.

### 2.2. Animals and Experimental Design

All animal care and experimental procedures were approved by the Animal Care and Use Committee of Nanjing Agricultural University (SYXK2019-0066) and conducted in accordance with the standard of the Animal Care and Use Guidelines of Nanjing Agricultural University (EACUGC2018-01). Twenty-four weaned male piglets (Duroc × Landrace × Large White) with an initial weight of 7.37 ± 0.52 kg (mean ± SEM) were weaned at 21 days of age and were randomly distributed into 4 experimental groups (*n* = 6), including the control group (CON), LPS group (LPS), CPN + LPS group (CPN-LPS) and CME + LPS group (CME-LPS). The piglets were selected from 10 litters of equal growth status. Piglets in the CON and LPS groups were fed basal diets, while piglets in the CPN-LPS and CME-LPS groups were fed the basal diets supplemented with CME or CPN (the concentration of cordycepin was 1 mg/kg). The whole experimental process lasted for 24 days, including a 3-day adaptation period and a 21-day trial period. Throughout the 24 days, all the piglets had free access to feed and water. The basal diet formula was consistent with our previous research [11].

### 2.3. Liver Injury Model

On the last day of the trial period, piglets in the LPS, CPN-LPS and CME-LPS groups were given intraperitoneal injections of LPS at a dose of 100 µg/kg body weight. The LPS, sourced from *Escherichia coli* O55:B5, was provided by Sigma Chemical Inc, St. Louis, MO, USA. At the same time, the piglets in the control group (CON) also received the same volume of saline as the treatment group. Four hours after the injection, the blood and liver samples were collected.

### 2.4. Sampling

Prior to sampling, all piglets were fasted. Four hours after the injection of LPS or saline, blood samples were drawn from the anterior vena cava using a blood collection vessel without any anticoagulants, following which piglets were anesthetized and euthanized by intravenous injection of 50 mg/kg pentobarbital sodium solution. The blood samples were centrifuged at 3000× *g* for 10 min at 4 °C to separate the serum, which was subsequently used for serum biochemical tests. The liver was excised, and the central portion was preserved in a 4% paraformaldehyde solution for morphological observation. Additionally, a portion of the liver was collected in a sterile cryopreservation tube and rapidly placed in liquid nitrogen, then transferred to −80 °C for subsequent analysis.

### 2.5. Serum Biochemical Tests and Liver Indicators

Serum aspartate transaminase (AST) and alanine aminotransferase (ALT) levels were measured using assay kits from Nanjing Jiancheng Technology Co., (Nanjing, China). C-reactive protein (CRP) and haptoglobin (HP) levels in serum and liver were measured by enzyme-linked immunosorbent assay (ELISA, Jiancheng Bioengineering Institution, Nanjing, China).

### 2.6. Liver HE Staining

The liver tissue was placed in 4% paraformaldehyde solution at 4 °C for fixation for 48 h, then the tissue was immersed in paraffin three times for 40 min each, followed by embedding. The liver tissue was then cut into 2 µm sections and stained using the haematoxylin and eosin (HE) staining method. The histopathological evaluation was performed under the ECLIPSE E100 microscope (Nikon Instruments Inc., Shanghai, China). The liver injury score was determined according to a method established in a previous study [22].

### 2.7. Hepatic Transcriptome Analysis

A sufficient quantity of liver tissue was harvested to extract total RNA, which was then purified using magnetic beads coated with poly T oligomers. RNA integrity was assessed using the RNA Nano 6000 Assay Kit of the Bioanalyzer 2100 system (Agilent Technologies, CA, USA). Quantification and purification were conducted using the AMPure XP system, and the quality of the library was evaluated on an Agilent Bioanalyzer 2100 system. After cluster generation, the library preparations were sequenced on an Illumina Novaseq platform and 150 bp paired-end reads were generated. Differentially expressed genes with a fold change (FC) of >2 or a FC of <0.5 and a *p*-Value of <0.05 were selected using DESeq2 R package edge (Version 3.0.3). Volcano plots were used to filter DEGs of interest which based on log_2_FC and −log_10_(*p*-Value) of metabolites by ggplot2 in R language. Differentially expressed genes were selected by Kyoto Encyclopedia of Genes and Genomes (KEGG) fold enrichment with cluster Profiler R package (Version 3.0.3).

### 2.8. Untargeted Hepatic Metabolome Analysis

Liver tissue (100 mg) was ground to powder by addition of liquid nitrogen and then resuspended in pre-cooled 80% methanol for homogenisation. Subsequently, the samples were subjected to an ice water bath, centrifuged and the supernatant was diluted with LC-MS grade methanol to a final concentration of 53% methanol. The sample was then transferred to a new Eppendorf tube and centrifuged at 15,000× *g* for 20 min at 4°C. The supernatant was injected into the LC-MS column. Finally, the supernatant was injected into the LC-MS/MS analysis system. The LC-MS/MS raw data files generated by UHPLC-MS/MS were processed using the Compound Discoverer 3.3 (CD3.3, ThermoFisher, MA, USA) to perform peak alignment, peak picking, and quantitation for each metabolite. These metabolites were annotated using the KEGG database (https://www.genome.jp/kegg/pathway.html, accessed on 25 August 2023). Principal components analysis (PCA) and Partial least squares discriminant analysis (OPLS-DA) were performed at metaX (ve. The metabolites with VIP > 1 and *p*-Value < 0.05 and FC ≥ 1.2 or FC ≤ 0.83 were considered to be differential metabolites. Heatmaps and differential metabolism maps were created using the Pheatmap package and correlation analysis with the R package. The functions of these metabolites and metabolic pathways were investigated using the KEGG database, and the metabolic pathways of differential metabolites were enriched. The enrichment of metabolic pathways was considered statistically significant when the metabolic pathway *p*-Value was 0.05.

### 2.9. Statistical Analysis of Data

The statistical analysis of experimental data was conducted using SPSS 27.0.1. All data were presented as mean ± SEM. One-way ANOVA analysis and Tukey’s multiple comparison tests were used for comparisons among four groups, and Student’s *t*-test for pairwise comparisons. *p* < 0.05 was considered a statistically significant difference, * *p* < 0.05, ** *p* < 0.01, *** *p* < 0.001. All graphs were produced using GraphPad Prism 8 software. Correlation analysis was performed using Pearson, and a strong significant correlation was considered when *p* < 0.05 and R > 0.8.

## 3. Results

### 3.1. Ameliorative Effects of Cordyceps Militaris Extract and Cordycepin on LPS-Induced Acute Liver Injury

**Figure 1:**
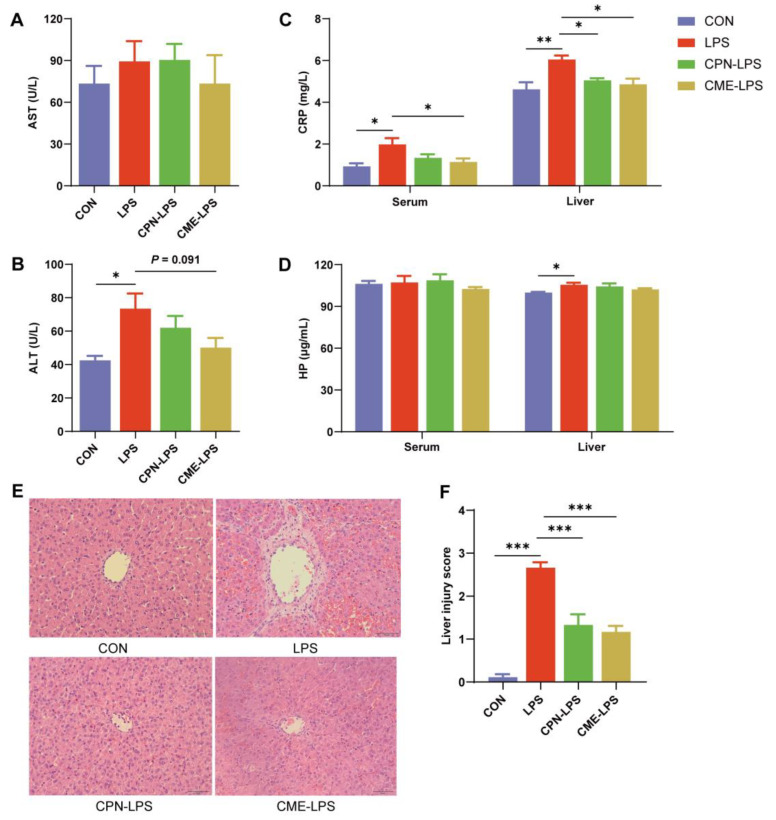
The alleviation of *Cordyceps militaris* extract and cordycepin on liver injury induced by LPS in piglets. CON: control group, LPS: LPS injection group, CPN-LPS: cordycepin (CPN) supplementation + LPS injection group, CME-LPS: *Cordyceps militaris* extract (CME) supplementation + LPS injection group. (**A**) Aspartate aminotransferase (AST) level in serum. (**B**) Alanine aminotransferase (ALT) level in serum. (**C**) C-reactive protein (CRP) level in serum and liver. (**D**) Haptoglobin (HP) level in serum and liver. (**E**) The histopathological changes in liver tissues of H&E staining (200×), scale bar with 100 μm. (**F**) The injury score of liver. Data were expressed as mean ± SEM (n = 6). Statistical significance was determined by One-way ANOVA analysis. * *p* < 0.05, ** *p* < 0.01, *** *p* < 0.001.

In order to ascertain whether liver injury was induced by LPS and whether *Cordyceps militaris* extract and cordycepin had a protective effect against this injury, we measured serum liver function indexes (Figure 1A,B) and acute phase protein (APP, Figure 1C-D) of serum and liver tissue. The results showed that a single LPS injection significantly increased the levels of serum ALT, CRP, and liver CRP and HP in piglets (*p* < 0.05), indicating liver injury and acute reactions in the LPS group when compared with the CON group. However, compared with the LPS group, the serum and liver CRP levels in the CME-LPS group were significantly decreased (*p* < 0.05), and the serum ALT level in the CME-LPS group demonstrated a downward trend (*p* = 0.091, Figure 1B). The liver CRP level in the CPN-LPS group was significantly lower than the LPS group (Figure 1C). In addition, HE staining of liver sections from piglets in the control group revealed normal liver morphology and structure, with liver cells exhibiting orderly arrangement and intact nuclei (Figure 1E). In contrast, the LPS group exhibited severe liver injury, with dilated liver sinuses, red blood cell filling, nuclear pyknosis and inflammatory cell infiltration. The liver tissue morphology of the CPN-LPS and CME-LPS groups exhibited a significant improvement in these lesions (Figure 1E,F).

### 3.2. Transcriptomic Analysis of Piglet Liver

**Figure 2:**
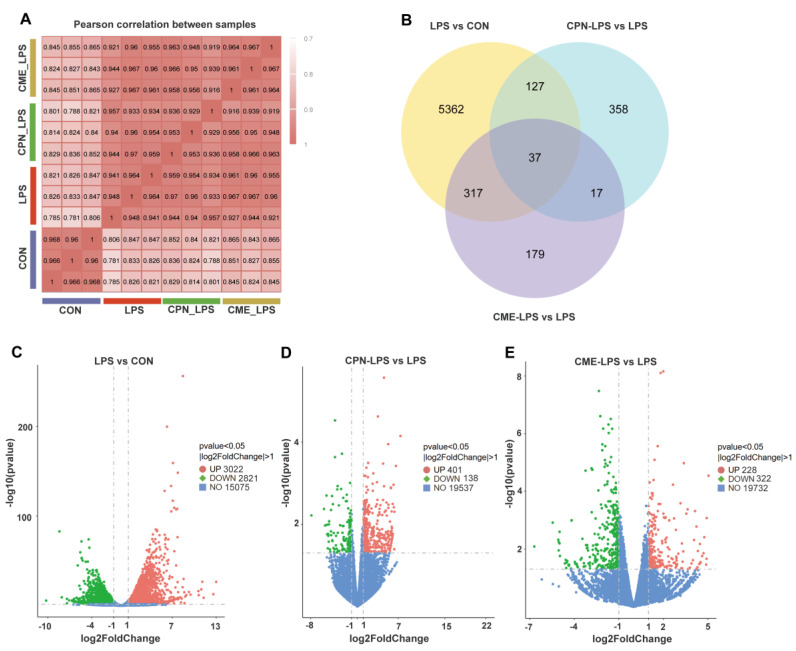
Transcriptome analysis of piglet liver (*n* = 3). CON: control group, LPS: LPS injection group, CPN-LPS: cordycepin (CPN) supplementation + LPS injection group, CME-LPS: *Cordyceps militaris* extract (CME) supplementation + LPS injection group. (**A**) Correlation analysis of gene expression of samples. (**B**) Venn diagram. Volcanic diagram of LPS vs. CON (**C**), CPN-LPS vs. LPS (**D**) and CME-LPS vs. LPS (**E**).

As shown in Figure 2A, the sample correlations ranged from 0.78 to 0.97, demonstrating good within-group repeatability. In order to discover as many potential results as possible for preliminarily exploring the mechanism of the attenuating role of CPN or CME on LPS-induced liver injury, this study identified DEGs with *p* < 0.05 and FC > 2 or FC < 0.5, referring to the screening criteria of previous studies [23,24,25]. The Venn plot showed only 37 overlapped DEGs in three comparison pairs (Figure 2B). Therein, in the volcano plot, compared to the CON group, the LPS group identified 5843 DEGs, with 3022 genes showing upregulated expression and 2821 genes showing downregulated expression (Figure 2C). Compared to the LPS group, 401 and 138 genes in CPN-LPS were upregulated and downregulated, respectively (Figure 2D), while 228 and 322 genes in CME-LPS were upregulated and downregulated, respectively (Figure 2E).

**Figure 3:**
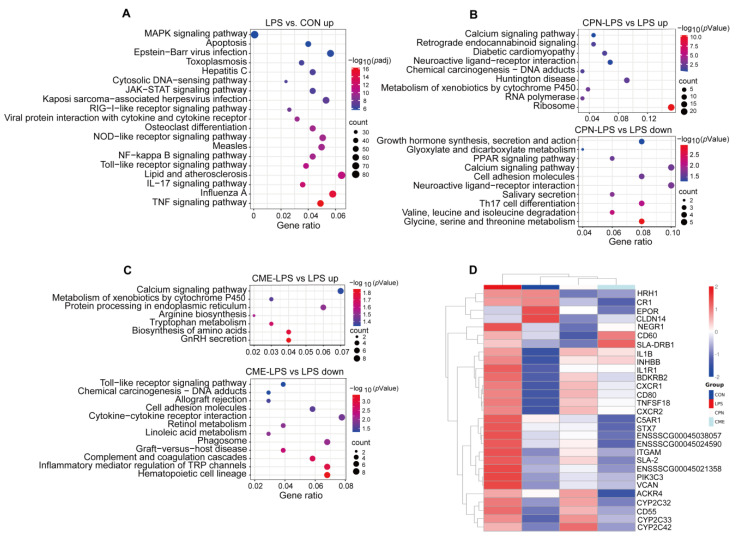
Pathway enrichment analysis of transcriptome. CON: control group, LPS: LPS injection group, CPN-LPS: cordycepin (CPN) supplementation + LPS injection group, CME-LPS: *Cordyceps militaris* extract (CME) supplementation + LPS injection group. (**A**) Top 20 KEGG enrichment analysis of upregulated DEGs in LPS vs. CON. KEGG analysis of upregulated and downregulated DEGs of CPN-LPS vs. LPS (**B**) and CME-LPS vs. LPS (**C**). (**D**) Heatmap of immune-related genes in the KEGG enrichment pathway of CPN-LPS vs. LPS and CME-LPS vs. LPS.

Then, KEGG enrichment analysis was performed on the DEGs in each pair of comparisons. Compared with CON, LPS treatment upregulated many pathways involved in the inflammatory response, such as cytokine-cytokine receptor interaction, tumour necrosis factor (TNF) signaling pathway, IL-17 signaling pathway and nucleotide-binding oligomerization domain (NOD)-like receptor signaling pathway, etc. (Figure 3A). As shown in Figure 3B, the significantly downregulated pathways in CPN-LPS compared with LPS mainly included glycine, serine and threonine metabolism, valine, leucine and isoleucine degradation, cell adhesion molecules and calcium signalling pathway, while the significantly downregulated pathways in CME-LPS compared with LPS mainly included inflammatory mediator regulation of TRP channels, complement and coagulation cascades, and phagosome and cytokine-cytokine receptor interaction (Figure 3C). And for the upregulated pathways, ribosome and metabolism of xenobiotics by cytochrome P450 were significantly enriched in CPN-LPS compared with LPS (Figure 3B), glycine, serine and threonine metabolism and biosynthesis of amino acids were significantly enriched in CME-LPS compared with LPS (Figure 3C). 29 DEGs were identified and clustered to generate a heatmap by selecting immune-related genes in the KEGG pathway that changed significantly in the comparisons CPN-LPS vs. LPS and CME-LPS vs. LPS (Figure 3D).

### 3.3. Metabolomics Analysis of Piglet Liver

**Figure 4:**
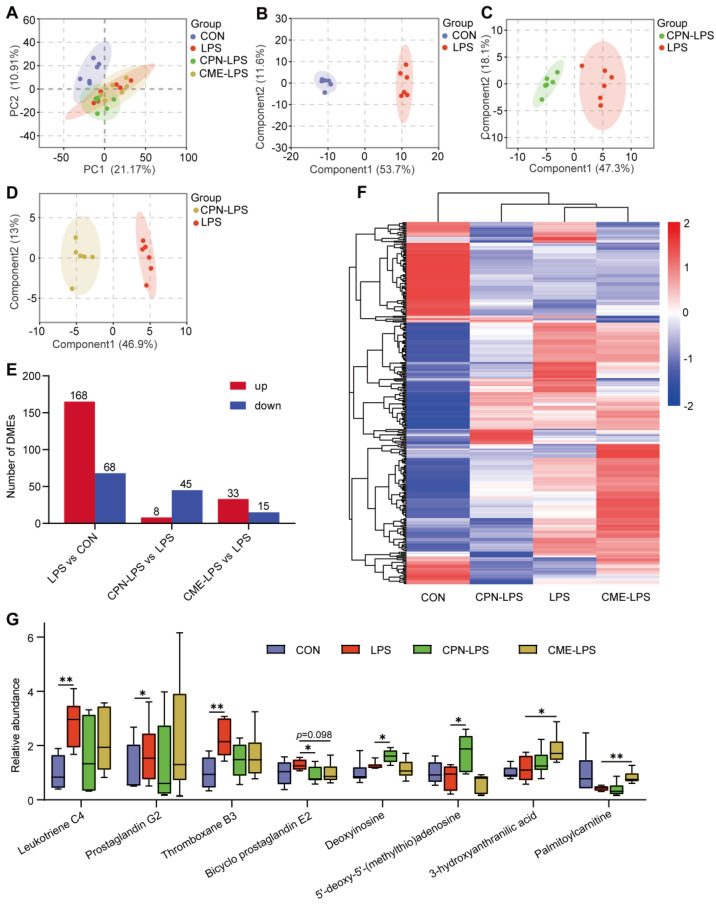
Metabolome analysis of piglet liver (*n* = 6). CON: control group, LPS: LPS injection group, CPN-LPS: cordycepin (CPN) supplementation + LPS injection group, CME-LPS: *Cordyceps militaris* extract (CME) supplementation + LPS injection group. (**A**) PCA plot. OPLS-DA plot of LPS vs. CON (**B**), CPN-LPS vs. LPS (**C**) and CME-LPS vs. LPS (**D**). (**E**) Changes of liver DEMs. (**F**) Cluster heatmap of differential metabolites. (**G**) Relative abundance of selected potential metabolites biomarkers. Statistical significance was determined by Student’s *t*-test. * *p* < 0.05, ** *p* < 0.01.

As shown in Figure 4A, the results of the principal component analysis (PCA) indicated that the control group was significantly separated from the other three groups. Subsequently, orthogonal partial least squares discriminant analysis (OPLS-DA) model was constructed for the purpose of comparing two groups, with varying degrees of separation between LPS vs. CON, CPN-LPS vs. LPS and CME-LPS vs. LPS (Figure 4B–D). According to the screening criteria with a FC greater than 1.2 or less than 0.83 and a *p*-Value less than 0.05, a total of 236, 53 and 48 differential metabolites were identified in the comparisons LPS vs. CON, CPN-LPS vs. LPS and CME-LPS vs. LPS, respectively (Figure 4E). Among them, 168 and 68 DEMs were up- and down-regulated in LPS compared with CON, 8 and 45 DEMs were up- and down-regulated in CPN-LPS compared with LPS and 33 and 155 DEMs were up- and down-regulated in CME-LPS compared with LPS. A total of 313 DEMs were identified in three comparison combinations and displayed in the heatmap (Figure 4F). As shown in Figure 4G, after LPS treatment, the relative abundance of potential biomarkers increased significantly, including biomarkers for leukotriene C4 (LTC4), prostaglandin G2 (PGG2) and thromboxane B3 (TXB3) (*p* < 0.05). However, the relative abundance of bicyclo-prostaglandin E2 (bicyclo-PGE2) decreased in CPN-LPS and CME-LPS compared to LPS. In addition, CPN-LPS significantly increased deoxyinosine (DI) and 5′-deoxy-5′-(methylthio)adenosine (MTA) and CME-LPS significantly increased 3-hydroxyanthranilic acid (3HAA) and palmitoylcarnitine compared to LPS (*p* < 0.05).

**Figure 5:**
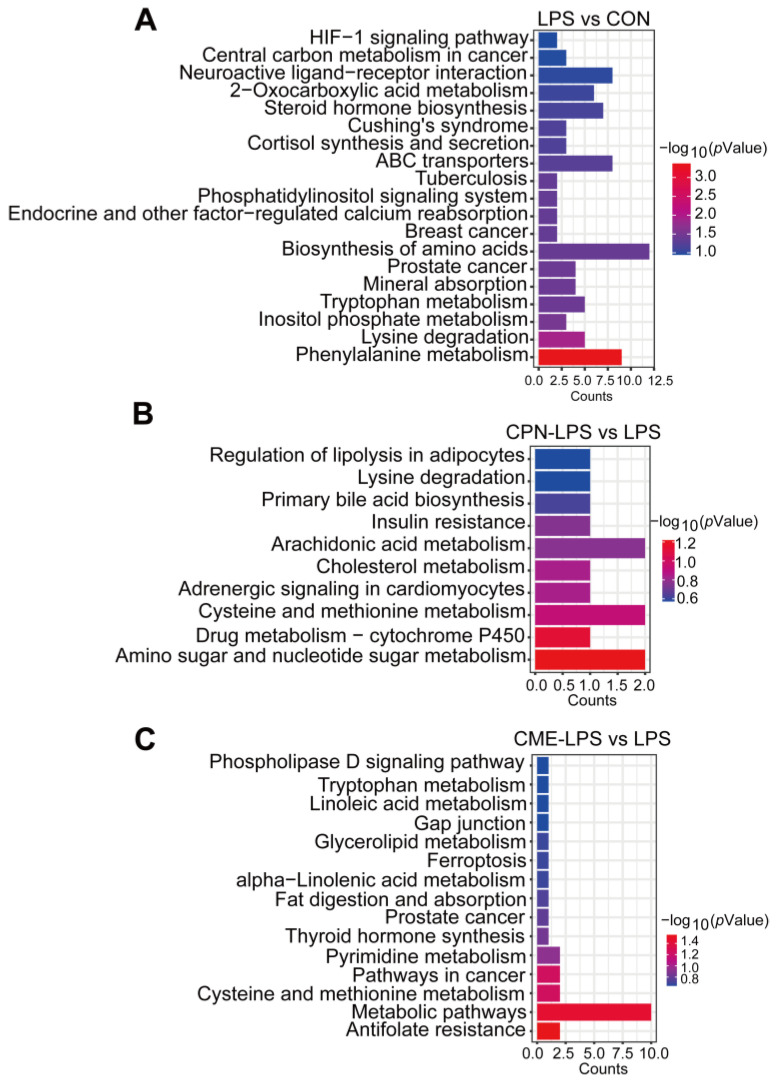
KEGG enrichment analysis of LPS vs. CON (**A**), CPN-LPS vs. LPS (**B**) and CME-LPS vs. LPS (**C**).

Finally, KEGG enrichment analysis showed that the DEMs between LPS and CON were significantly enriched in phenylalanine metabolism, lysine degradation, innositol phosphate metabolism and tryptophan metabolism (Figure 5A). The DEMs between CPN-LPS and LPS were mainly enriched in amino sugar and nucleotide sugar metabolism, amino sugar and nucleotide sugar metabolism, cholesterol metabolism and arachidonic acid metabolism, among others (Figure 5B). In the comparison between CME-LPS and LPS, metabolic pathways, cysteine and methionine metabolism, glycerolipid metabolism and fat digestion and absorption were enriched (Figure 5C).

### 3.4. Combination of Transcriptomic and Metabolomic Analysis on the Protective Effect of Liver Injury

**Figure 6:**
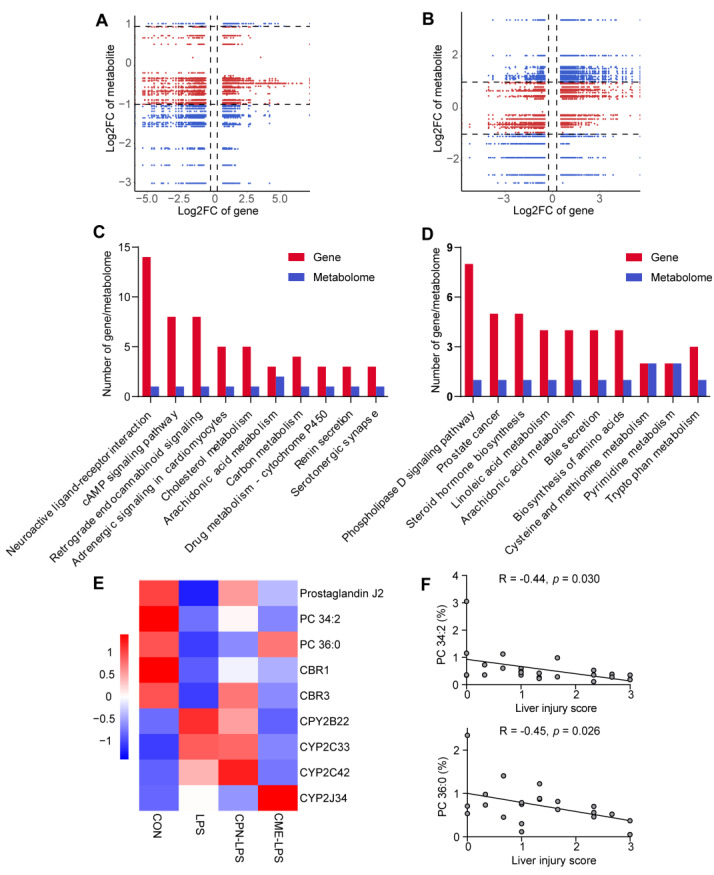
Integration analysis of transcriptome and metabolome. CON: control group, LPS: LPS injection group, CPN-LPS: cordycepin (CPN) supplementation + LPS injection group, CME-LPS: *Cordyceps militaris* extract (CME) supplementation + LPS injection group. The nine-quadrant plots show the correlation between DEGs and DEM in CPN-LPS vs. LPS (**A**) and CME-LPS vs. LPS (**B**). Pearson correlation of differential genes and metabolites (*n* = 3). *p* < 0.05, R > 0.8. Each point represents DEGs and DEMs pairs, blue and red points indicate DEGs and DEMs pairs and DEGs and non-DEM pairs, respectively. The absolute value corresponding to the dashed lines on the horizontal and vertical axes is 1, which signifies that the FC of the genes or metabolites is 2 or 0.5. Quadrants one to nine are ordered from left to right and top to bottom. Quadrants 1, 3, 7, and 9 with blue points represent DEGs and DEMs. Quadrants 4 and 6 with red points represent DEGs and non-DEMs. Quadrants 2 and 8 represent non-DEGs and DEMs, while quadrant 5 represents non-DEGs and non-DEMs. The top 10 KEGG common pathways with the highest number of DEGs and DEMs in CPN-LPS vs. LPS (**C**) and CME-LPS vs. LPS (**D**) mapped to the KEGG. (**E**) Heatmap of DEMs and DEGs of arachidonic acid metabolism. (**F**) Correlation analysis between relative abundance of PC 34:2 and PC 36:0 and liver injury score.

As shown in Figure 6A,B, correlation analyses were performed on DEMs and DEGs. The DEMs and DEGs shown in quadrants 3 and 7 were positively correlated, while the DEMs and DEGs shown in quadrants 1 and 9 were negatively correlated. DEMs and DEGs pairs were more in quadrant 7 in CPN vs. LPS (Figure 6A), and DEMs and DEGs pairs were more in quadrant 3 in CME vs. LPS (Figure 6B), indicating that differentially expressed genes mostly regulate metabolites positively. By co-enriching DEMs and DEGs in KEGG, it was found that neuroactive ligand receptor interaction, cAMP signalling pathway and cholesterol metabolism were co-enriched in CPN-LPS vs. LPS (Figure 6C), whereas phospholipase D signalling pathway, steroid hormone biosynthesis and linoleic acid metabolism were co-enriched in CPN-LPS vs. LPS (Figure 6D). Notably, the arachidonic acid metabolism pathway was found to be present in both groups CPN-LPS vs. LPS and CME-LPS vs. LPS. Of them, CPN supplementation restored the decreased abundance of prostaglandin J2 (PGJ2) and PC 34:2, and CME supplementation increased the abundance of PC 36:0 (Figure 6E). The transcriptome results found that CME supplementation decreased the expression of *CPY2B22*, *CYP2C33* and *CYP2C42* and increased the expression of *CYP2J34*. Additionally, CPN supplementation increased the expression of *CBR1* and *CBR3*. The metabolites PC 34:2 and PC 36:0 showed a significant negative correlation with the liver injury score (Figure 6F, *p* < 0.05).

## 4. Discussion

In recent years, there has been a notable increase in research focused on the potential of nutritional interventions for the treatment of liver damage resulting from immune stress [3,16,26,27]. LPS can cause acute liver injury by inducing oxidative stress and an inflammatory response, and cordycepin has been reported to have many benefits for the body, including the treatment of many liver diseases. In this study, we demonstrated that CPN and CME could prevent acute liver injury caused by LPS. By integrating transcriptomics and metabolomics, we investigated the metabolites and genes associated with LPS-induced liver injury and elucidated the therapeutic mechanisms of CPN and CME in alleviating LPS-induced acute liver injury. Our research results suggest that the hepatoprotective effects of CPN and CME may be due to the regulation of immune-related genes and metabolites. These findings may contribute to a better understanding of the potential of CPN and CME in the prevention of acute liver injury.

Liver injury caused by LPS injection manifests as acute reactions, while cordycepin with anti-inflammatory and antioxidant activity can alleviate liver damage [14]. AST and ALT are the most sensitive indicators of liver function tests, and their serum levels are elevated in cases of liver cell damage [28,29]. In this study, we observed that a single injection of LPS resulted in a significant increase in serum ALT level in piglets when compared to the control group. This finding is consistent with a previous study that have demonstrated an increase in serum AST, ALT and ALP (alkaline phosphatase) levels and a damage in liver morphology on day 1 following LPS injection [3]. Similarly, the liver HE staining chart of the LPS group in this study showed severe liver damage, but this damage was found to be improved in pigs supplemented with CME or CPN in advance. In the event of infection or tissue damage, the body is capable of initiating acute phase reactions and the production of acute phase proteins (APP) in a relatively short period of time [30]. C-reactive protein (CRP) and haptoglobin (HP) are APP that are synthesised by liver cells [11]. CRP responds rapidly to adverse stimuli, with serum levels potentially increasing by thousands of times, depending on the nature and severity of the disease [31]. HP also exhibits a marked increase when under stress [32]. The present study demonstrated that the serum and liver CRP and serum HP levels were significantly increased in the LPS group compared to the control group, indicating that liver injury caused by LPS injection manifests as acute reactions. In addition, CME or CPN supplementation can reduce LPS-induced CRP levels in piglets. Our previous study demonstrated that the LPS group exhibited an increase in serum levels of pro-inflammatory factors (IL-1β and IL-8), whereas supplementation with CPN or CME exhibited an increase in serum levels of the anti-inflammatory factor IL-10 [11]. Therefore, *Cordyceps militaris* extract and cordycepin can alleviate LPS induced acute liver injury.

To investigate the protective mechanism of CME or CPN against liver injury in piglets injected with LPS, a liver transcriptome analysis was conducted. The results showed that, compared with CON, LPS treatment upregulated many pathways involved in the inflammatory response, which also proved a possible pathway for LPS to cause severe liver injury and was consistent with previous research [33]. CME supplementation has, to some extent, restored these pathways, including inflammatory mediator regulation of TRP channels, complement and coagulation cascades, and phagosome, cytokine-cytokine receptor interaction and cell adhesion molecules. TRP channels, which belong to the superfamily of nonselective calcium-permeable cation channels, act as multiple cellular injury receptors, detecting various environmental stimuli and modulating autoimmunity and inflammation by regulating intracellular calcium levels [34]. Studies have demonstrated that nonylphenol-induced pancreatitis results in the upregulation of inflammatory mediator regulation of TRP channels [35]. Excessive complement activation and coagulation can result in liver injury and act as proliferative factors of the inflammatory process [36]. Cytokine-cytokine receptor interaction is involved in pathological processes such as inflammatory injury, and cytokines are key regulators and mobilisers of the inflammatory host defence [37]. Triclosan (TCS) has been reported to cause upregulation of cytokines and cytokine receptor interaction pathways in mouse liver injury [38]. Overexpression of cell surface adhesion molecules can promote the adhesion and migration of inflammatory cells to the intima, leading to endothelial dysfunction and vascular damage [39]. Notably, the cell adhesion molecules pathway was found to be significantly downregulated in both CPN-LPS vs. LPS (including genes *CD80*, *SLA-2*, *ITGAM* and *VCAN*) and CME-LPS vs. LPS (including genes *SLA-DRB1*, *CD6*, *CLDN14* and *NEGR1*). Cell adhesion molecules are crucial for normal homeostasis and various pathological diseases [40]. *CD80*, a co-stimulatory molecule expressed by antigen-presenting cells (APCs) such as macrophages, is upregulated on activated macrophages and promotes the secretion of pro-inflammatory cytokines [41]. Integrin alpha M (*ITGAM*) plays a role in the processes of cell adhesion, migration and phagocytosis [42]. The expression of *VCAN* and *SLA2* may have pro-inflammatory effects [43,44]. Cordycepin has been extensively reported to mitigate acute liver injury through the reduction of inflammation [13,14,45,46]. In this study, we also found that the upregulation of inflammation-related genes induced by LPS was reversed in the CME or CPN groups. For instance, the gene *IL1R1*, which activates the inflammatory signaling pathway, is significantly downregulated in both CPN and CME [47]; the gene *IL1B*, which encodes the widely recognised pro-inflammatory cytokine interleukin-1β, amplifies the immune response [48]. In summary, the transcriptomics analysis demonstrated that supplementing with CME or CPN mitigates LPS-induced liver injury, in part, through pathways related to inflammation.

Metabolites represent the ultimate products of biological processes, and thus metabolomic analysis can directly elucidate the mechanisms by which CPN and CME alleviate LPS-induced liver injury [19]. The results demonstrated that 165 and 68 DEMs in LPS were upregulated and downregulated, respectively, in comparison to CON. Therein, we focused on identifying inflammatory markers and acute phase reactants to predict markers of acute liver injury [49]. In this study, it was observed that treatment with LPS significantly increased the abundance of potential disease biomarkers for leukotriene C4 (LTC4), prostaglandin G2 (PGG2) and thromboxane B3 (TXB3). LTC4 and PGG2 are both pro-inflammatory substances that are derived from arachidonic acid (AA) [50,51]. Increasing evidence suggests that LTC4 is involved in a variety of liver injuries, and LTC4 synthase expression has been found to be upregulated in D-GalN/LPS-induced rat liver injury [52]. Besides, the ischemia–reperfusion (I/R) injury in rat liver is associated with an abnormal increase in LTC4 production [53]. PGG2 can increase inflammation, pain, and fever [54]. TXB3 also has pro-inflammatory activity, but with less inflammatory [55]. However, the levels of these metabolites were not significantly different in the CPN-LPS and CME-LPS groups compared to CON. We also found that CPN and CME supplementation reduced the metabolic levels of bicyclo prostaglandin E2 (bicyclo-PGE2) in LPS-treated piglets. Bicyclo-PGE2 is a product associated with the pro-inflammatory effect of the stable degradation of PGE2, and elevated levels have been reported in the context of infection [51,56]. In contrast, significant increases were observed in deoxyinosine (DI) and 5′-deoxy-5′-(methylthio)adenosine (MTA) from CPN-LPS and 3-hydroxyanthranilic acid (3HAA) and palmitoylcarnitine from CME-LPS compared to the LPS group. DI, an anti-inflammatory factor, inhibits LPS-induced inflammation both in vitro and in vivo [57]. Palmitoylcarnitine attenuates liver I/R injury via the Nrf2/HO-1 axis [58]. MTA and 3HAA have antioxidant properties [59,60]. The above results suggest that CPN or CME supplementation to induce changes in liver metabolites may help to ameliorate LPS-induced liver injury.

The integrated transcriptome and metabolome analysis revealed alterations in DEGs and DEMs within the co-enriched pathways. In these co-enriched pathways, arachidonic acid metabolism co-exists in CPN and CME treatments. The arachidonic acid metabolism is linked to the progression of many liver diseases, with AA and its derivatives influencing immune regulation and the inflammatory response [61,62]. Among these, our results revealed that CPN supplementation restored the decreased abundance of prostaglandin J2 (PGJ2) and PC 34:2. Similarly, CME supplementation increased the abundance of PC 36:0. It has been reported that the metabolites PGJ2 and 15-deoxy-PGJ2 of prostaglandin D2 (PGD2) possess anti-inflammatory properties, which are mediated by a negative feedback loop [63]. PC is a vital component of cell membranes and has anti-inflammatory and hepatocyte-protective properties that can be employed to prevent and treat liver diseases [64]. The liver has a high demand for PC and disruption of the integrity of liver cell membranes can lead to cell apoptosis, inflammation and the development of liver disease [14,65]. In the DEGs and DEMs identified in the co-enrichment pathway, the correlation analysis between the relative abundance of PC 34:2 and PC 36:0 and the liver injury score demonstrated a significant negative correlation. This may be the reason why we observed in the sections that CPN and CME supplementation restored the morphology of the liver cells. AA is primarily metabolised into various metabolites via the cyclooxygenase (COX), lipoxygenase (LOX), and cytochrome P450 (CYP-450) metabolic pathways [65]. The transcriptome results found that CME supplementation decreased the expression of *CPY2B22*, *CYP2C33* and *CYP2C42* and increased the expression of *CYP2J34*, which are involved in arachidonic acid metabolism. Additionally, CPN supplementation increased the expression of two carbonyl reductases (CR), *CBR1* and *CBR3*. CR represents a fundamental enzyme-based defense mechanism against oxidative stress [66]. Therefore, we speculate that DEGs and DEMs in arachidonic acid metabolism, especially PC 34:2 and PC 36:0 which are associated with morphological recovery of hepatocytes, play an important role in supplementing CPN and CME to attenuate LPS-induced liver injury.

There were some limitations to this study. While the study succeeded in identifying key pathways and metabolites, it was regrettable that there was a dearth of results pertaining to the mutual regulation between metabolites and genes involved in liver injury recovery pathways. A more comprehensive analysis may yield deeper insights. While the study suggested potential mechanisms through multi-omics analysis, direct mechanistic studies were required to confirm the roles of identified genes and metabolites in the context of liver injury. Furthermore, the short duration of the supplementation may not fully reflect the long-term effects of CPN or CME on liver health. These limitations indicate a need for further research to validate and expand upon the current findings.

## 5. Conclusions

This study demonstrated that CPN or CME supplementation significantly reduced C-reactive protein level and improved liver tissue pathology following LPS injection. The transcriptomic results showed that LPS treatment upregulated many inflammation-related pathways, whereas CPN or CME supplementation downregulated some inflammation-related pathways. CPN-LPS downregulated cell adhesion molecules, while CME-LPS downregulated inflammatory mediator regulation of TRP channels, complement and coagulation cascades and cytokine-cytokine receptor interaction. The metabolomic results showed that CPN or CME supplementation reduced disease biomarkers of bicyclo-prostaglandin E2, increased levels of deoxyinosine and 3-hydroxyanthranilic acid. The combined transcriptome and metabolome indicated that the arachidonic acid metabolism pathway of CPN or CME supplementation may play a pivotal role in protecting against liver injury. CPN and CME may attenuate LPS-induced liver injury in piglets by modulating the expression of genes and metabolites associated with inflammatory processes. The findings will contribute to a better understanding of the potential of CPN and CME in the alleviation of acute liver injury and provide insights into nutritional interventions to prevent liver disease.

## Data Availability

The original contributions presented in the study are included in the article, further inquiries can be directed to the author.
